# Safety and feasibility of the ISO-LPR protocol (isotretinoin, laser, and polydeoxyribonucleotide) in severe nodulocystic acne: A case report

**DOI:** 10.1016/j.jdcr.2026.03.018

**Published:** 2026-03-19

**Authors:** Rafael Rodrigo Crisanto de Oliveira

**Affiliations:** Midas Clínica, Ipueiras, Ceará, Brazil

**Keywords:** acne vulgaris, isotretinoin, PDRN, Q-Switched Nd:YAG, laser therapy, wound healing

## Introduction

Severe nodulocystic acne presents a dual therapeutic challenge involving rapid inflammatory control and prevention of permanent dermal scarring. Oral isotretinoin remains the gold standard therapy due to its sebosuppressive, anti-inflammatory, and comedolytic properties. However, its concomitant use with energy-based devices has historically been discouraged due to concerns regarding impaired wound healing and increased risk of hypertrophic scar formation, leading to the conventional recommendation of a 6-m post-treatment washout period before laser procedures.^1^^,^^2^

Recent evidence challenges this paradigm, suggesting that concomitant laser therapy may be safe and potentially beneficial when appropriately applied during isotretinoin treatment.^3^ Nonetheless, structured multimodal protocols integrating systemic therapy, photoacoustic modulation, and regenerative stimulation remain insufficiently characterized.

This report describes the clinical evolution of a patient treated with the ISO-LPR Protocol, a multimodal approach designed to simultaneously address inflammation, sebaceous gland activity, follicular obstruction, and regenerative repair during ongoing isotretinoin therapy.

## Case report

**Fig 1:**
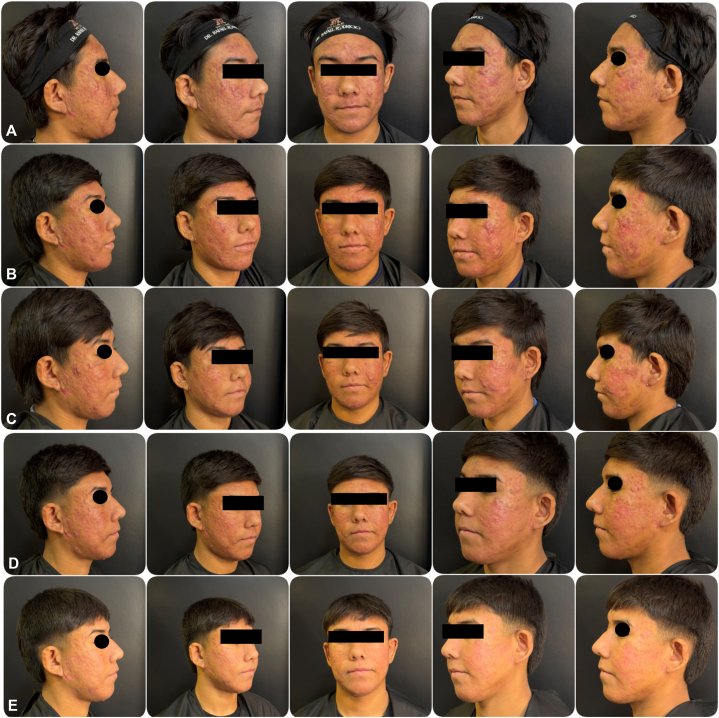
Sequential clinical progression of a patient treated with a multimodal protocol (oral isotretinoin, Black Peel, and topical PDRN). **(A)** Baseline showing severe nodulocystic acne. **(B**-**D)** Months 1–4, demonstrating gradual reduction of inflammatory lesions, improvement of postinflammatory erythema, and early texture regularization. **(E)** Five-month outcome showing complete clearance and absence of adverse scarring. Dates: A: May 31, 2025; B: Jul 23, 2025; C: Aug 22, 2025; D: Oct 23, 2025; E: Nov 21, 2025.

A 16-year-old male presented with severe nodulocystic acne characterized by extensive inflammatory papules, pustules, and deep nodules involving the entire face (Fig 1, *A*). No relevant medical history or comorbidities were present.

The patient weighed 60 kg and was initiated on oral isotretinoin in June 2025 at a dose of 20 mg/day (0.33 mg/kg/d), with a planned cumulative target dose of approximately 120 mg/kg. Isotretinoin was administered continuously without interruption and remained ongoing at the time of follow-up on January 26, 2026.

The ISO-LPR Protocol consisted of the following:

1. Systemic control:

Continuous oral isotretinoin 20 mg/d without interruption from June 2025 through January 26, 2026. At the time of follow-up, the cumulative dose reached approximately 4800 mg, corresponding to 80 mg/kg, with treatment ongoing toward the planned cumulative target.

2. Photoacoustic modulation:

Monthly sessions of carbon-assisted Q-Switched Nd:YAG 1064 nm laser (Black Peel) were performed from June through December 2025, totaling 7 sessions, using a fluence of 2 J/cm^2^.

Topically applied carbon particles function as exogenous chromophores that absorb laser energy and generate rapid photoacoustic and photomechanical shockwaves. These effects promote superficial exfoliation, reduce follicular obstruction, and may contribute to reduction in Cutibacterium acnes colonization through sebaceous modulation and follicular clearance, while minimizing thermal injury to surrounding tissues.^4^

Unlike ablative or fractional lasers, Q-Switched Nd:YAG laser does not create permanent microchannels but induces transient photomechanical effects that may increase epidermal and follicular permeability, potentially facilitating enhanced topical penetration.

3. Regenerative drug delivery:

Immediately following each laser session, 1 mL of topical 1.6% polydeoxyribonucleotide (PDRN) enriched with growth factors (IGF, TGF-β3, and Tβ4) (PDRN Factors, La Cutanée, Brazil) was applied.

PDRN is a DNA-derived polymer that activates adenosine A2A receptors, promoting anti-inflammatory signaling, fibroblast proliferation, angiogenesis, and extracellular matrix remodeling, thereby facilitating regenerative tissue repair rather than fibrotic healing.^5^^,^^6^

Application immediately after laser exposure leveraged transient increases in epidermal permeability induced by photoacoustic effects, facilitating enhanced topical penetration.

4. Adjunctive care

Home care was restricted to barrier repair agents (ceramides, hyaluronic acid, and panthenol) and strict photoprotection. Written informed consent was obtained from the patient and his legal guardians for the publication of this case report and any accompanying images.

### Clinical course

**Fig 2:**
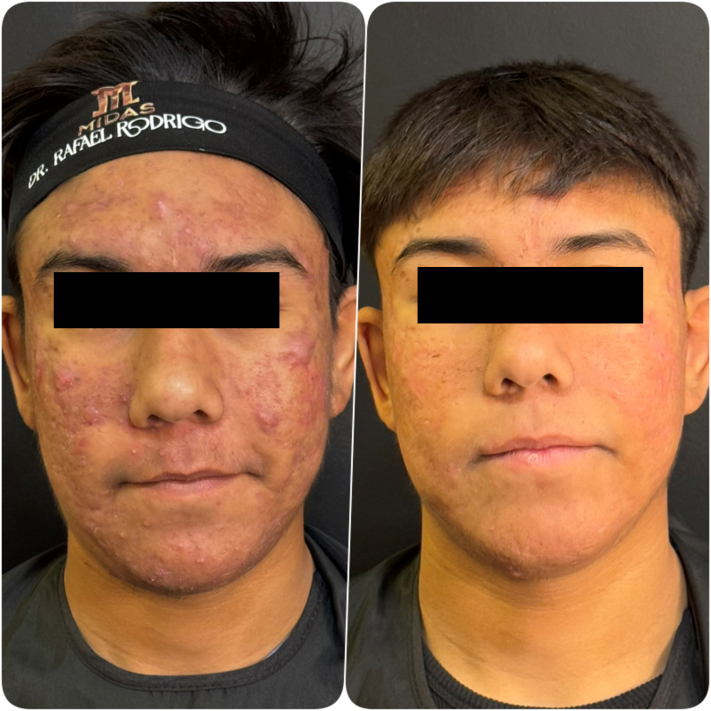
Clinical evolution of severe acne vulgaris. (Left) Baseline presentation (May 31, 2025) showing extensive inflammatory papules, pustules, and nodules on the face. (Right) Post-treatment appearance (November 21, 2025) demonstrating significant reduction in inflammatory lesions and overall improvement in skin texture after 5 months of treatment.

Progressive clinical improvement was observed throughout treatment (Figs 1 and 2). Early reduction of inflammatory lesions and improvement in postinflammatory erythema were evident during the first months of therapy.

By the end of the fifth month of therapy (November 2025), while still receiving isotretinoin and prior to reaching the cumulative target dose, complete clearance of active inflammatory lesions was achieved, accompanied by visible improvement in skin texture and remodeling of preexisting atrophic scars.

Monthly laser sessions were continued through December 2025, completing a total of 7 sessions.

**Fig 3:**
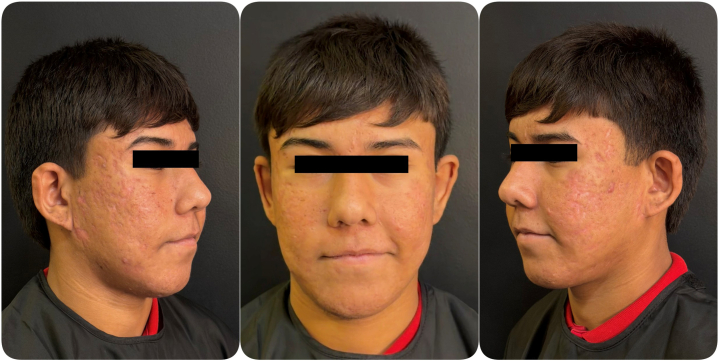
Extended follow-up (January 26, 2026) demonstrating sustained clearance and continued dermal remodeling during ongoing isotretinoin therapy.

Follow-up evaluation performed on January 26, 2026, after approximately 8 months of uninterrupted isotretinoin therapy and 7 laser sessions, demonstrated sustained clinical remission, continued dermal remodeling, and absence of hypertrophic scarring, delayed wound healing, or atypical tissue response (Fig 3). Isotretinoin therapy remained ongoing at the time of this follow-up.

No paradoxical inflammatory flares occurred.

Adverse effects were limited to mild cheilitis and one episode of herpes labialis, which resolved with standard antiviral therapy.

## Discussion

This case challenges the longstanding caution regarding laser use during isotretinoin therapy.

Complete inflammatory clearance occurred at a cumulative isotretinoin dose of approximately 50 mg/kg, prior to reaching conventional cumulative targets. This observation suggests that the ISO-LPR Protocol may have contributed to accelerated inflammatory resolution. However, given the known efficacy of isotretinoin monotherapy, a synergistic effect cannot be definitively established from a single case.

Meta-analyses have demonstrated that combining isotretinoin with laser therapy does not increase adverse events and may enhance treatment efficacy.^3^

The carbon-assisted Q-Switched Nd:YAG laser produces photoacoustic effects that improve follicular clearance and sebaceous modulation while avoiding thermal injury.^4^ Unlike ablative lasers, this modality induces transient permeability changes rather than permanent microchannels.

PDRN contributes regenerative benefits through activation of adenosine A2A receptors, modulation of inflammatory pathways, stimulation of fibroblast activity, and promotion of extracellular matrix remodeling.^5^^,^^6^

Importantly, no evidence of impaired wound healing, hypertrophic scarring, or delayed re-epithelialization was observed despite repeated laser exposure during active isotretinoin therapy, supporting emerging evidence challenging historical contraindications.^3^

Although most evidence supporting PDRN efficacy involves intradermal administration, topical application following laser-induced photomechanical effects may enhance penetration. However, the extent of dermal delivery through this mechanism remains to be fully elucidated.

## Conclusion

This case demonstrates that the ISO-LPR Protocol may represent a safe and effective multimodal strategy for accelerating inflammatory resolution and promoting regenerative repair during ongoing isotretinoin therapy. Sustained clinical remission and absence of adverse wound healing outcomes following multiple laser sessions support the feasibility of combining carbon-assisted Q-Switched Nd:YAG laser and topical PDRN with systemic isotretinoin. Prospective controlled studies are warranted to validate these findings.

## Conflicts of interest

None disclosed.
